# Elucidating the aluminum storage mechanism in cobalt sulfide cathode materials for advanced batteries

**DOI:** 10.3389/fchem.2025.1633529

**Published:** 2025-07-15

**Authors:** Ruiyuan Zhuang, Yongqing Li, Junhong Wang, Jianfeng Zhan, Jiangnan Yan, Yaru Chen, Wenhui Mo, Jun Zhang

**Affiliations:** ^1^ School of Mechanical and Electrical Engineering, Jiaxing Nanhu University, Jiaxing, China; ^2^ School of Materials Science and Engineering, Jiangsu University, Zhenjiang, Jiangsu, China; ^3^ College of Biosystems Engineering and Food Science, Zhejiang University, Hangzhou, Zhejiang, China

**Keywords:** aluminum-ion batteries, Co_9_S_8_ cathode, pseudomorphic substitution, reaction kinetics, electrochemical performance, density functional theory

## Abstract

Rechargeable aluminum-ion batteries (AIBs), as novel energy storage systems featuring low-cost, high-energy density, and superior safety, demonstrate promising potential as a next-generation battery technology. However, the lack of high-performance cathode materials remains a critical barrier to practical implementation. In this study, highly crystalline cobalt sulfide (Co_9_S_8_) nanoparticles were synthesized using a one-step hydrothermal method and systematically evaluated their electrochemical performance and energy storage mechanisms in AIBs. Structural characterization revealed that while the synthesized material maintained high crystallinity, it formed agglomerates during the synthesis process that induced severe electrode polarization and limited ion diffusion kinetics. Electrochemical analysis demonstrated a reversible capacity of 48 mAh g^−1^ after 500 cycles at a current density of 100 mA g^−1^, indicating moderate cycling stability. DFT calculations with Bader charge analysis provided atomic-scale insights, revealing that Al^3+^ preferentially occupies Co. lattice sites through a pseudo-isomorphic substitution mechanism, exhibiting a 52.5% lower formation energy compared to S-site substitution. This work establishes critical correlations between morphological characteristics and electrochemical performance while proposing a novel cation substitution mechanism for energy storage. These findings provide fundamental insights for designing high-kinetics transition metal sulfide cathodes and advance the development of practical multivalent-ion battery systems.

## 1 Introduction

Amidst the accelerating global carbon neutrality strategy, electrochemical energy storage technologies have emerged as pivotal solutions to reconcile the intermittency of renewable energy sources with grid stability, owing to their high energy conversion efficiency and versatile application scenarios. Current mainstream electrochemical storage systems include lithium-ion batteries (LIBs), sodium-ion batteries (SIBs), zinc-ion batteries (ZIBs), and supercapacitors (Cao et al., 2025; Xie et al., 2025; Wei and Chen, 2025; Maurya et al., 2025; Jing et al., 2025; Tao et al., 2025; Ma J. et al., 2024). Among these, LIBs dominate the market due to their high energy density and mature commercialization. However, the scarcity of lithium resources (0.0065% crustal abundance), uneven geographical distribution, and environmental concerns have driven urgent demands for alternative energy storage systems (Gao et al., 2024; Wang et al., 2024). Aluminum-ion batteries (AIBs) have garnered significant attention as compelling candidates for large-scale energy storage, capitalizing on aluminum’s exceptional crustal abundance (8%), exceptional theoretical capacity (2,980 mAh g^−1^), and cost-effectiveness. Notably, AIBs exhibit intrinsic safety (dendrite-free characteristics) and environmental compatibility, positioning them as promising solutions for grid-scale storage and electric vehicles (Gupta et al., 2024; Yang et al., 2024; Yang and Huang, 2024).

Despite their potential, practical AIBs applications face multifaceted challenges. In aqueous systems, issues such as aluminum corrosion, hydrogen evolution side reactions, and passivation layer formation severely degrade cycling stability (Yu et al., 2024; Stephanie et al., 2024). More critically, cathode material limitations remain the primary bottleneck for non-aqueous AIBs. Since the pioneering work by Lin et al. (2015), who established the AlCl3/EMImCl ionic liquid electrolyte system, research on non-aqueous AIBs has expanded rapidly. However, non-aqueous AIBs practical performance are hindered by sluggish electrochemical kinetics attributed to the low electrode potential of aluminum and the large ionic radius involved in electrode reactions. Recent efforts have explored diverse cathode materials, including metal oxides, transition metal chalcogenides (TMCs), carbon-based materials, and others (Gupta et al., 2024; Yang et al., 2024; Yang and Huang, 2024). Carbon-based materials (graphite, graphene) demonstrate high discharge voltages and cycling stability but suffer from limited capacity due to AlCl_4_^–^ intercalation mechanisms, yielding only one-third of the theoretical capacity achievable via Al^3+^ intercalation. Transition metal oxides offer high specific capacity but face severe Coulombic interactions and lattice diffusion barriers caused by high charge density of Al^3+^. In contrast, TMCs exhibit superior promise for AIBs cathodes owing to their multi-electron redox activity, wide interlayer spacing, and low electronegativity, enabling high theoretical capacities and improved Al^3+^ diffusion kinetics (Ma D. et al., 2024; Wu et al., 2019; Huang et al., 2024; Liu et al., 2021).

Among TMCs, cobalt sulfide (Co_9_S_8_) has attracted particular interest due to its unique crystal structure and electrochemical properties. Recent studies highlight its versatility in energy storage: Bai et al. engineered core-shell Co_9_S_8_ nanospheres via hydrothermal and annealing methods, demonstrating exceptional sodium-ion storage performance (Zhang et al., 2018). Feng et al. developed Co_9_S_8_ hollow boxes with dual-open ends via solvothermal synthesis, achieving remarkable rate capability and cycling stability in SIBs through pseudocapacitive effects (Yin et al., 2020). Zhang et al. developed an innovative hierarchical architecture of Co_9_S_8_-MoB MBene heterostructures. The precisely embedded Co_9_S_8_ nanoparticles within the interlayer spacing of MoB MBene frameworks endowed the composite with exceptional lithium storage properties, demonstrating superior rate capability and cycle stability as a high-performance cathode material for LIBs, outperforming state-of-the-art transition metal sulfide-based cathodes (Wang et al., 2025). Additionally, Zhang et al. fabricated a three-dimensional porous N-doped carbon composite embedded with MOF-derived Co_9_S_8_ nanoparticles (Co_9_S_8_@NPC) through a templating strategy coupled with *in situ* high-temperature pyrolysis. When evaluated as an anode material for potassium-ion batteries (KIBs), the Co_9_S_8_@NPC composite demonstrated superior electrochemical performance compared to previously reported metal sulfide-based anode materials for KIBs (Ma et al., 2022). Furthermore, reports exist on the application of Co_9_S_8_ in AIBs; however, a specific investigation into its electrode energy storage mechanism is lacking (Grindal and Azimi, 2024).

Herein, we present the comprehensive study of Co_9_S_8_ as an AIBs cathode, elucidating its electrochemical dynamics and atomic-scale Al^3+^ storage mechanisms. Nanocrystalline Co_9_S_8_ particles were synthesized via a one-step hydrothermal method, with structural and morphological properties characterized by XRD and SEM. Electrochemical performance was evaluated through CV, galvanostatic charge-discharge testing, and EIS. The Co_9_S_8_ cathode demonstrated notable cycling stability, retaining ∼48 mAh g^−1^ after 500 cycles at 100 mA g^−1^, with a minimal capacity decay rate of 0.04% per cycle. First-principles calculations revealed a distinctive “pseudo-isomorphic substitution” mechanism, where Al^3+^ preferentially occupies octahedral Co. sites in the Co_9_S_8_ lattice (binding energy: 0.92 eV). By elucidating structure-property relationships and Al^3+^ storage dynamics, this study provides critical insights for designing high-performance AIB cathodes and advances the development of cost-effective, durable energy storage systems.

## 2 Materials and methods

### 2.1 Material synthesis and characterization

**FIGURE 1:**
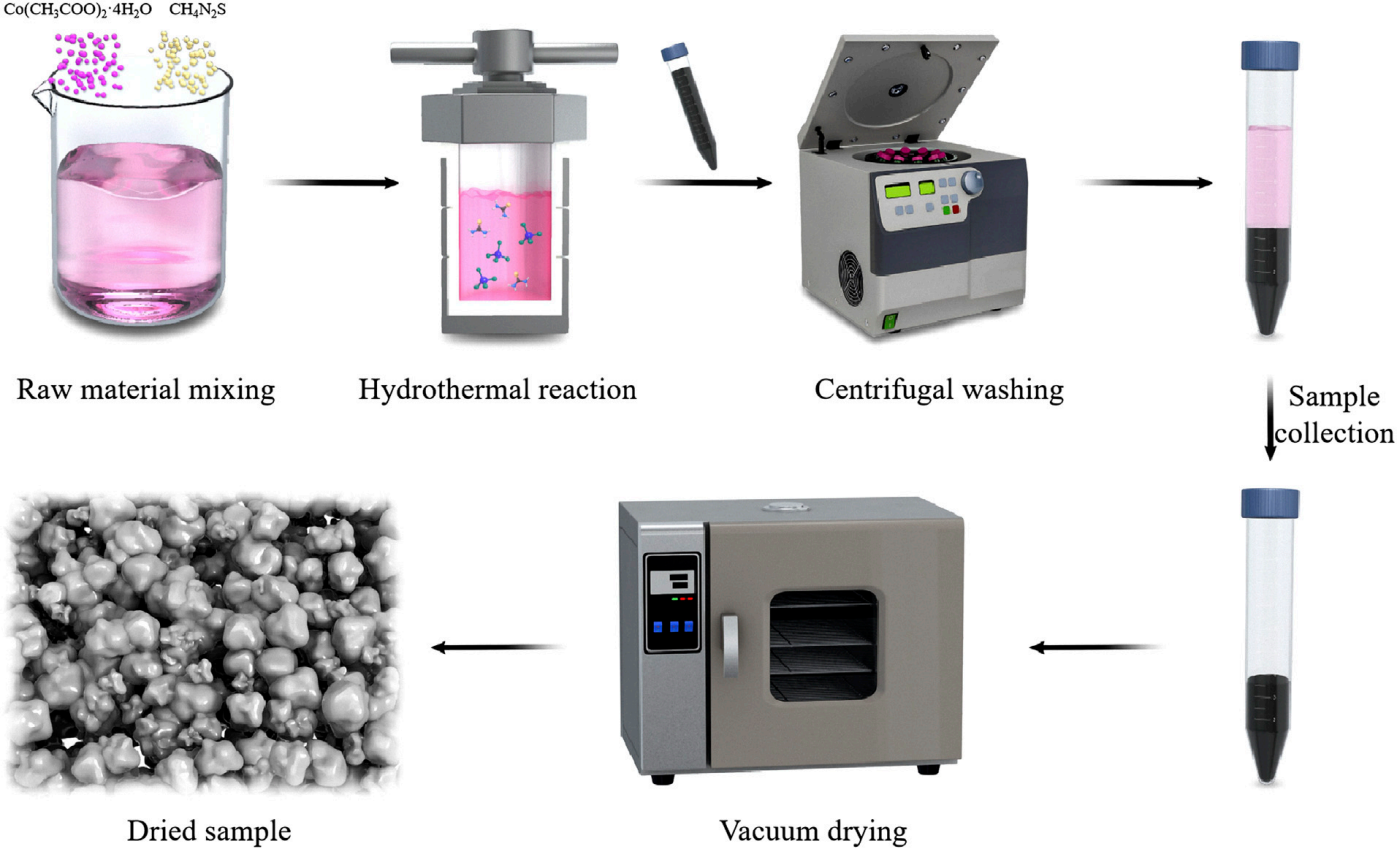
Schematic diagram of Co_9_S_8_ synthesis process.

The synthesis of Co_9_S_8_ followed a modified solvothermal method reported in the literature (Tian et al., 2022; Wang et al., 2025; Feng et al., 2016). Briefly, 5 mmol of cobalt (II) acetate tetrahydrate and 5 mmol of thiourea were separately dissolved in 60 mL of ethylene glycol under vigorous magnetic stirring at 600 rpm. The two solutions were then mixed and transferred into a Teflon-lined stainless-steel autoclave. The autoclave was heated at 200°C for 12 h and allowed to cool naturally to room temperature. The resulting black precipitate was collected and washed repeatedly with deionized water and absolute ethanol to remove impurities. Finally, the product was dried in a vacuum oven at 60°C for 12 h and stored in a desiccator for subsequent characterization. Figure 1 schematically presents the hydrothermal synthesis process of Co_9_S_8_ nanoparticles.

The crystallographic structure of the synthesized material was analyzed by X-ray diffraction (XRD, SmartLab 9 kW, Rigaku) with Cu-Kα radiation (λ = 1.5406 Å) in a 2θ range of 10°–80° at a scanning rate of 10° min^−1^ and a step size of 0.02°. Surface elemental composition and chemical states were characterized using X-ray photoelectron spectroscopy (XPS, Thermo Fisher Nexsa). The X-ray source used was a monochromatic Al-Kα source with an energy of 1,486.6 eV and a voltage of 12 KV. All binding energies were calibrated against the C 1s peak at 284.8 eV. Morphological features of the sample was observed using a JEOL JSM-7800F field emission scanning electron microscopy (SEM) operated at 5 kV.

### 2.2 Electrode and electrolyte preparation

The cathode slurry was prepared by homogeneously mixing the active material (Co_9_S_8_), conductive carbon, and binder in a predetermined mass ratio, pouring them into a mortar, adding an appropriate amount of anhydrous ethanol, and grinding them into a slurry at room temperature. In order to evenly mix the active material and conductive carbon, while ensuring that the adhesive is fully extended to better serve as a bridging agent, an appropriate amount of alcohol needs to be added during the grinding process to fully grind the electrode material and achieve the desired consistency. When the grinding is completed, place the sample on a roller mill, roll the slurry into thin sheets, and then dry the sample in a vacuum oven at 60 C for 12 h. After the sample is dried, use a punching machine to cut the thin sheets into round discs with 12 mm diameter, which are the electrode pieces for testing the battery. The ionic liquid electrolyte was synthesized by mixing 1-ethyl-3-methylimidazolium chloride ([EMIm]Cl) and AlCl3 at a molar ratio of 1:1.3. The preparation process was carried out in a glove box, and the prepared electrolyte was stored in the glove box.

### 2.3 Battery assembly and electrochemical testing

Pouch-type cells were utilized for electrochemical evaluations due to the high corrosivity of the ionic liquid electrolyte used in the experiment toward stainless steel. The tested battery components include cathode, separator, anode, and electrolyte. A Whatman GF/D glass fiber membrane and aluminum foil served as the separator and anode, respectively. For batteries assembly, the cathode, separator, and aluminum foil were stacked sequentially, encapsulated in an aluminum-laminated pouch, and injected with 250 μL of electrolyte. The pouch was vacuum-sealed using a thermal sealer. All procedures were conducted in the glove box to maintain strict moisture/oxygen control. Prior to testing, batteries were aged for 12 h to ensure complete electrolyte infiltration.

All constant current charge and discharge tests in the experiment were performed on a Neware electrochemical workstation within a voltage window of 0.1–1.8 V (vs. Al/AlCl_4_^–^). Cyclic voltammetry (CV) and electrochemical impedance spectroscopy (EIS) were conducted using a PARSTAT MCEIS instrument at Princeton Electrochemical Workstation. CV scans were executed at 0.5 mV s^−1^ over the same voltage range as constant current charge and discharge tests, while EIS measurements spanned frequencies from 100 kHz to 10 mHz with an AC amplitude of 10 mV. All tests were carried out at ambient temperature.

## 3 Results and discussion

**FIGURE 2:**
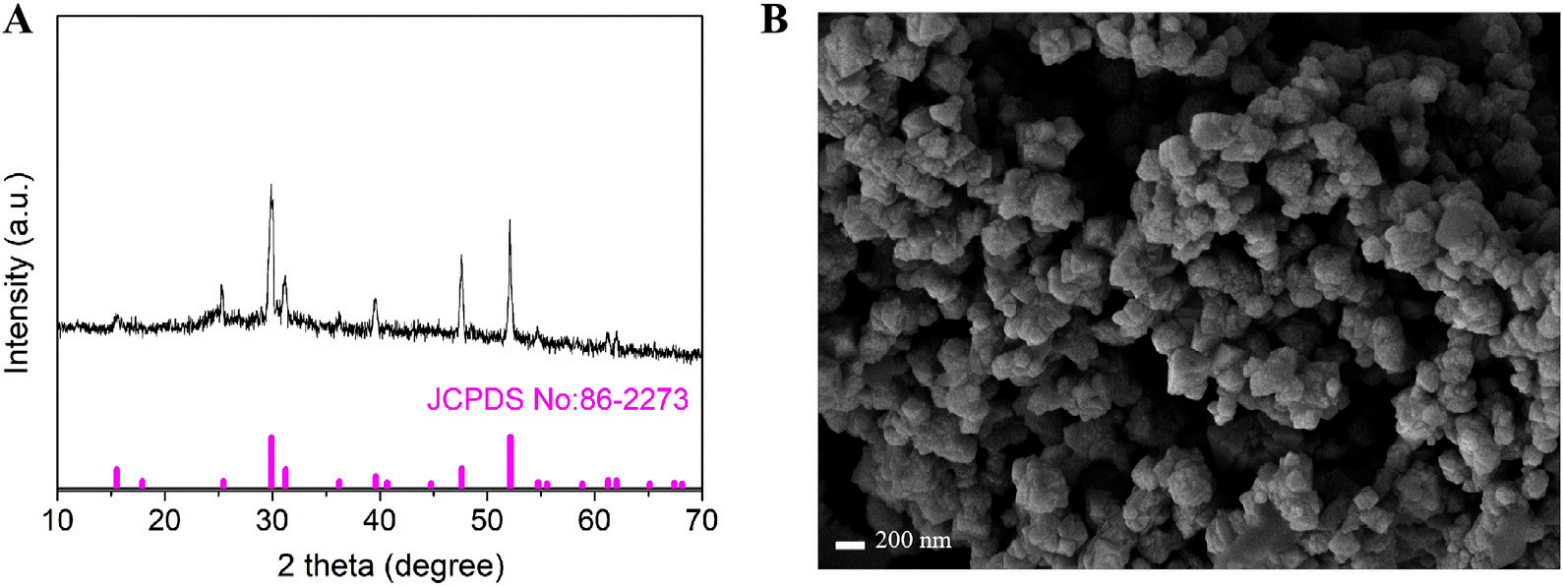
**(A)** XRD patterns of the obtained Co_9_S_8_, **(B)** SEM image of Co_9_S_8_.

In order to study the phase structure of the synthesized materials, X-ray diffraction (XRD) testing was performed on the sample. The results of XRD phase analysis on the sample are shown in Figure 2A. The analysis shows that the characteristic peaks located at 2θ = 15.4°, 25°, 4°29.8°, 31.2°, 36.2°, 39.5°, 44.7°, 47.6°, 52.1°, 61.2° and 62.1° correspond to the (111), (220), (311), (222), (400), (331), (420), (511), (440), (533) and (622) crystal planes of cubic Co_9_S_8_, respectively. All characteristic peak positions are highly consistent with the diffraction data of the JCPDS standard card (No.86-2273), and no diffraction signals of other impurities are detected (Tian et al., 2022; Feng et al., 2018). It is noteworthy that the full width at half maximum (FWHM) of each diffraction peak is less than 0.5, indicating that the prepared material has high crystallinity. The above structural characterization results fully demonstrate that the experiment successfully obtained Co_9_S_8_ nanomaterials with single phase and high crystallinity by optimizing the hydrothermal synthesis conditions. Figure 2B shows the scanning electron microscopy (SEM) image of Co_9_S_8_. It can be seen from the figure that the synthesized sample has an irregular shape, consisting of smaller nanoparticles clustered into larger structures. The exposed active sites of this large particle structure are reduced, leading to a decrease in the effective contact area between the electrode and the electrolyte, and a reduction in the interfacial reaction activity (Wang et al., 2015).

**FIGURE 3:**
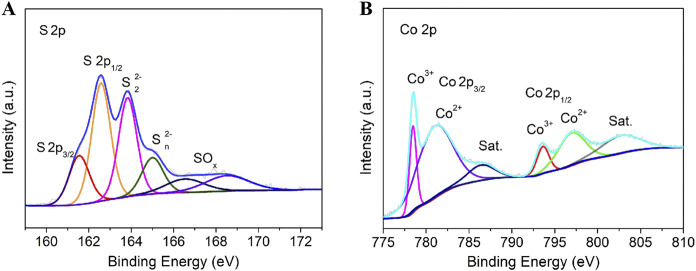
XPS spectra of **(A)** S 2p, **(B)** Co 2p.

The surface chemical composition and elemental electronic states of the material surface are systematically analyzed by X-ray photoelectron spectroscopy (XPS). By deeply analyzing the characteristics of each peak in the XPS spectrum, the chemical valence states and chemical bond properties of elements such as cobalt and sulfur in the sample can be inferred. The high-resolution spectrum of S 2p (Figure 3A) was deconvoluted into six distinct peaks after peak fitting. The double peaks at 161.5 eV and 162.6 eV correspond to the S 2p3/2 and S 2p1/2 orbitals of sulfide species (S2-), respectively (Feng et al., 2016; Feng et al., 2015). The characteristic peaks at 163.8 eV and 165.1 eV are attributed to disulfide (S2- 2) and polysulfide (S2- n) species, consistent with the crystal structure characteristics of Co_9_S_8_ (Tian et al., 2022; Zhou et al., 2015; Lv et al., 2024). Additional peaks at 166.6 eV and 168.6 eV indicate the presence of surface-oxidized sulfur species (SOx), likely due to partial oxidation of surface sulfur species during prolonged air exposure (Wang et al., 2025; Lv et al., 2024). The Co 2p high-resolution spectrum (Figure 3B) displays typical multiple splitting characteristics, mainly including a pair of Co 2p3/2, a pair of Co 2p1/2, and a pair of satellite peaks. Among them, the main peaks at 780.1 eV (Co 2p3/2) and 795.9 eV (Co 2p1/2) are assigned to Co^3+^ species, while the secondary peaks at 781.7 eV (Co 2p3/2) and 797.3 eV (Co 2p1/2) originate from Co^2+^ species (Tian et al., 2022; Wang et al., 2025; Zhou et al., 2015; Lv et al., 2024). In addition, satellite peaks observed at 786.7 eV and 802.2 eV further confirm the multivalent nature of cobalt, which is closely related to the complex electronic structure of Co_9_S_8_.

**FIGURE 4:**
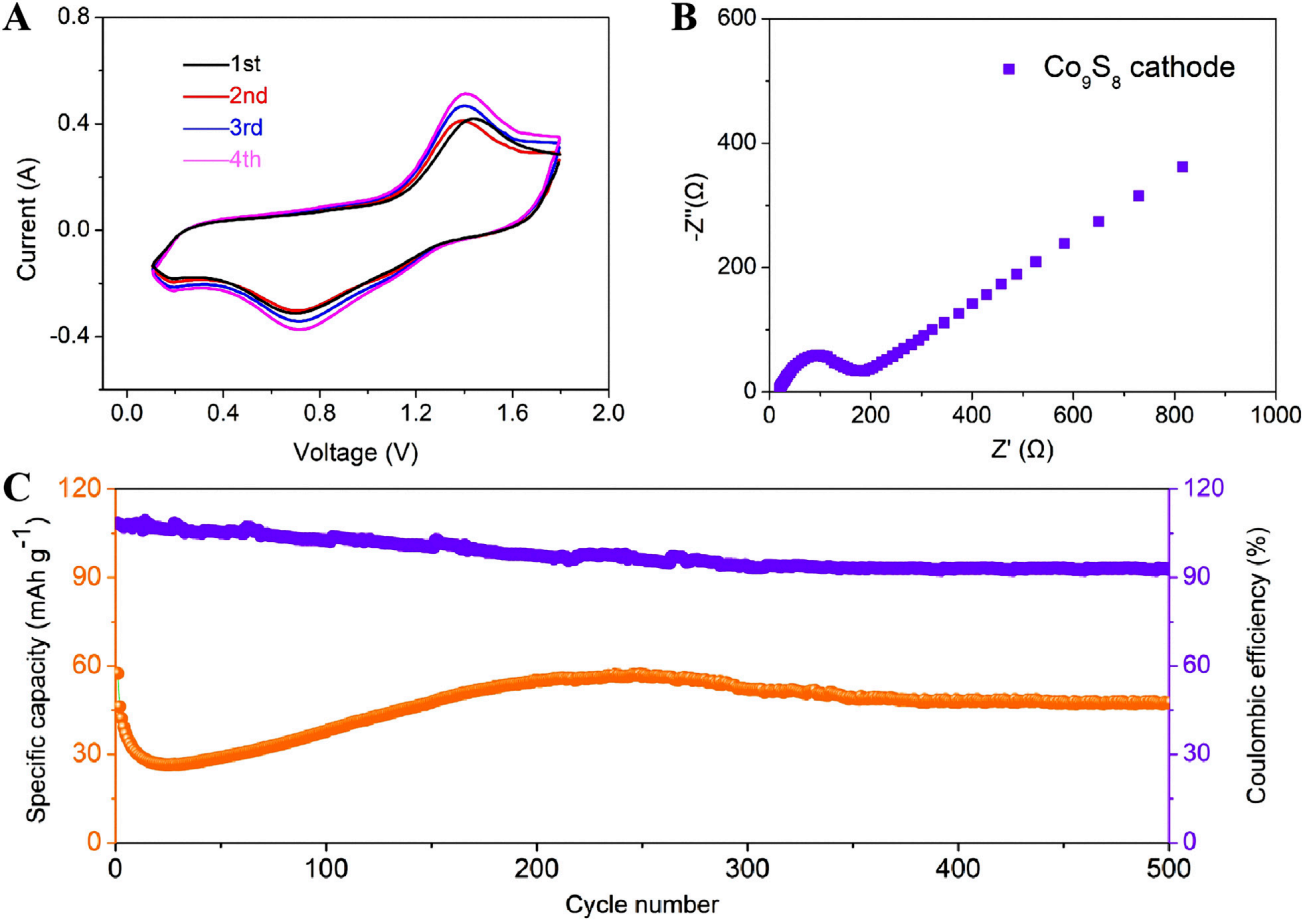
**(A)** CV curves of Co_9_S_8_ electrode at 0.5 mV s^−1^. **(B)** Nyquist plots of Co_9_S_8_ electrode. **(C)** Discharge-charge curves of Co_9_S_8_ electrode at 100 mA g^−1^.

To systematically evaluate the energy storage performance of Co_9_S_8_ electrode in AIBs, cyclic voltammetry (CV), electrochemical impedance spectroscopy (EIS), and constant current charge-discharge tests were conducted to comprehensively characterize its electrochemical behavior (Figure 4). The CV test was conducted at a scan rate of 0.5 mV s^−1^ within a voltage window of 0.1–1.8 V to elucidate the electrochemical activity of the electrode, with the corresponding curve shown in Figure 4A. It can be observed from the figure that all CV curves exhibit a pair of positive-negative peaks at approximately 1.41 V and 0.72 V, respectively. It is noteworthy that CV curves shows a significant polarization gap (ΔE = 0.69 V) in the electrode. This significant polarization is attributed to sluggish ion diffusion kinetics caused by the large particle size, which prolongs the Al^3+^ transport pathway from the surface to the bulk. Additionally, CV curves of the Co_9_S_8_ electrode shows broad and weak characteristic peaks, indicating that its electrochemical reaction kinetics are significantly inhibited, which may be related to insufficient active site exposure and structural constraints (Lou et al., 2020; Luo et al., 2023; Kope, 2011). Figure 4B shows the EIS spectrum of the Co_9_S_8_ electrode, and the fitted curve clearly demonstrates a high degree of agreement with the experimental data. As can be seen from the figure, the EIS spectrum consists of a semicircle and a diagonal line. Specifically, the slanted line in the low-frequency range reflects the ion diffusion capability in the electrolyte, while the semicircle in the high-frequency range corresponds to the electrode/electrolyte interface charge transfer resistance (Rct) (Panigrahi et al., 2024). For the EIS spectrum of the Co_9_S_8_ electrode, the Rct value is as high as 186 Ω, which is directly related to the reduction in the electrode/electrolyte contact area caused by its large particle morphology. The large angle between the straight line in the low-frequency region and the real axis in the EIS spectrum indicates that the ion diffusion process is relatively slow (Singh et al., 2017; Zeng et al., 2024). Figure 4C shows the long-term cycling performance curve of AIBs using Co_9_S_8_ electrodes under the working condition of 100 mA g^−1^. It should be noted that the Co_9_S_8_ electrode undergoes significant activation effects, which is similar to previous reports (Zhuang et al., 2021; Yang et al., 2019). The phenomenon of longer activation time for Co_9_S_8_ electrode may be attributed to the delayed accessibility of aluminum ions due to the maximum particle size of the surface-active substance, resulting in slower battery reaction kinetics (Zhaung et al., 2021; Elia et al., 2016). After activation, the electrode delivers a discharge capacity of about 65 mAh g^−1^. Then, it undergoes a slow decay process and eventually stabilizes after 350 cycles (∼48 mAh g^−1^). After that, the charge-discharge curve tends to a horizontal straight line, indicating that the electrode has good cycle stability. It is noteworthy that the Coulombic efficiency is stable at over 93%, indicating that the side reactions at the electrode interface are effectively suppressed.

**FIGURE 5:**
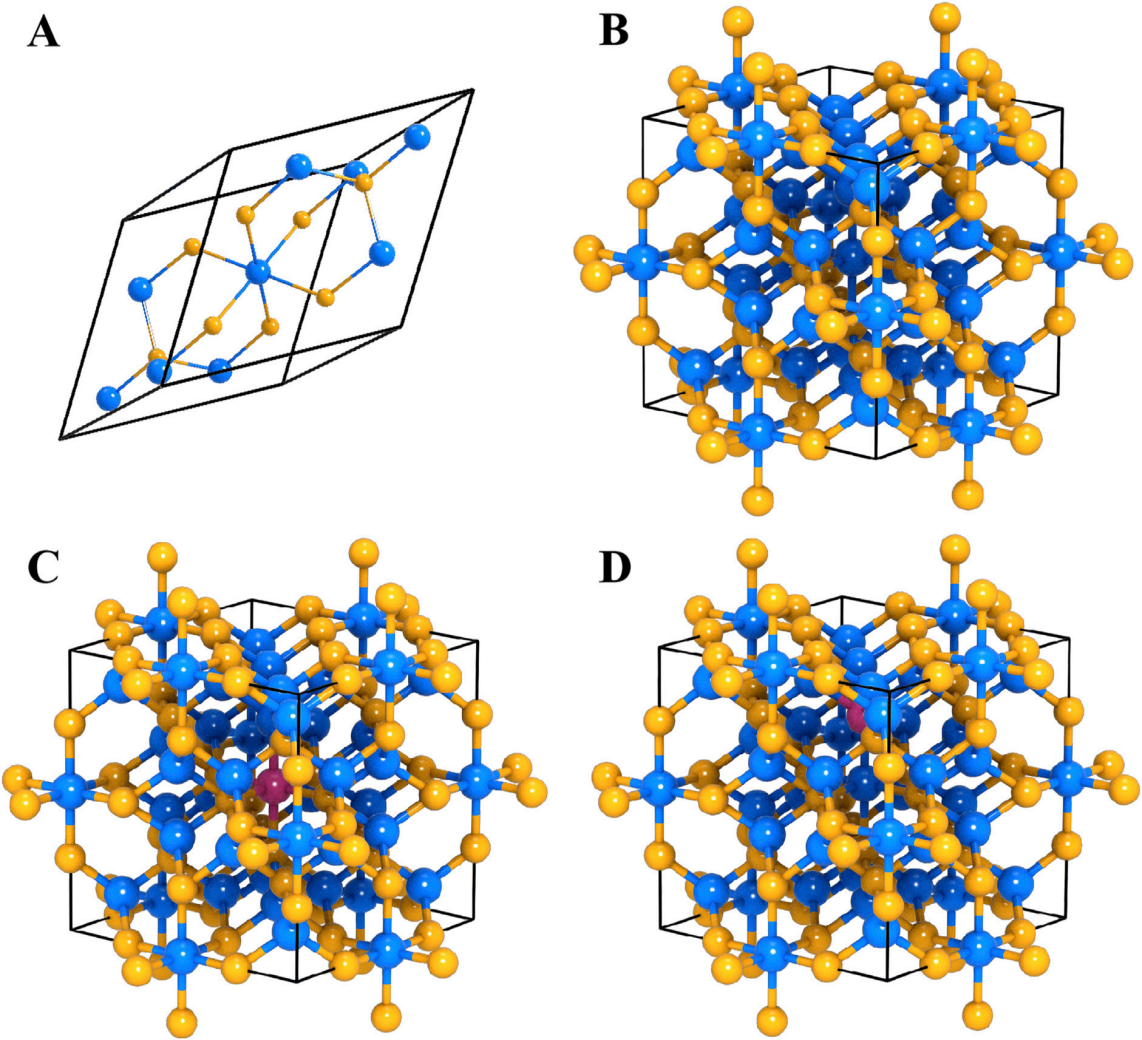
The crystal structure of Co_9_S_8_: **(A)** primitive cell, **(B)** supercell model; the crystal structure of Co_9_S_8_ with different substitutions: **(C)** Al substituted Co site, **(D)** Al substituted S site.

Although the electrode exhibits good cycle stability, its discharge capacity is still significantly lower than the practical application requirements of AIBs. To elucidate the intrinsic capacity limitations, this study finally combined density functional theory (DFT) calculations with multiscale simulation methods to systematically explore the crystal structure characteristics and Al^3+^ storage behavior of Co_9_S_8_. Figure 5 shows the crystal structure model of Co_9_S_8_ and the energetics analysis results of different Al^3+^ storage mechanisms. Figure 5A illustrates the structural configuration of the Co_9_S_8_ primitive unit cell, which consists of 17 atoms in total. Specifically, this configuration comprises one octahedrally coordinated Co metal atom (denoted as M(O)), eight tetrahedrally coordinated Co metal atoms (denoted as M(T)), two bridging sulfur atoms (S(l)), and six face-capping sulfur atoms (S(f)). These structural characteristics reveal that sulfur atoms engage in distinct coordination modes (bridging and face-capping) to construct a three-dimensional metal sulfide framework. This structural arrangement provides critical insights into understanding the physicochemical properties of the compound, establishing essential correlations between atomic coordination environments and macroscopic material behavior. Based on the VASP software package, the PAW-PBE functional was used to perform geometric optimization on the cubic phase of Co_9_S_8_ supercell (space group Fm3m, No. 225), and a 3 × 3 × 3 supercell model was constructed as shown in Figure 5B. After optimization, the lattice constant a = 9.842 Å, with a deviation of less than 0.3% from the benchmark value in the Materials Project database, verifying the reliability of the computational method. Structural analysis showed that the optimized Co_9_S_8_ supercell exhibited a typical cobalt sulfide structure, with 3D ion channels composed of octahedral interstices and tetrahedral vacancies providing a topologically adapted transport pathway for Al^3+^ diffusion. There was a significant mismatch between this size and the diameter of Al^3+^ ions, and the spatial confinement effect of this structure may be a key factor contributing to the kinetic retardation of ion diffusion.

**FIGURE 6:**
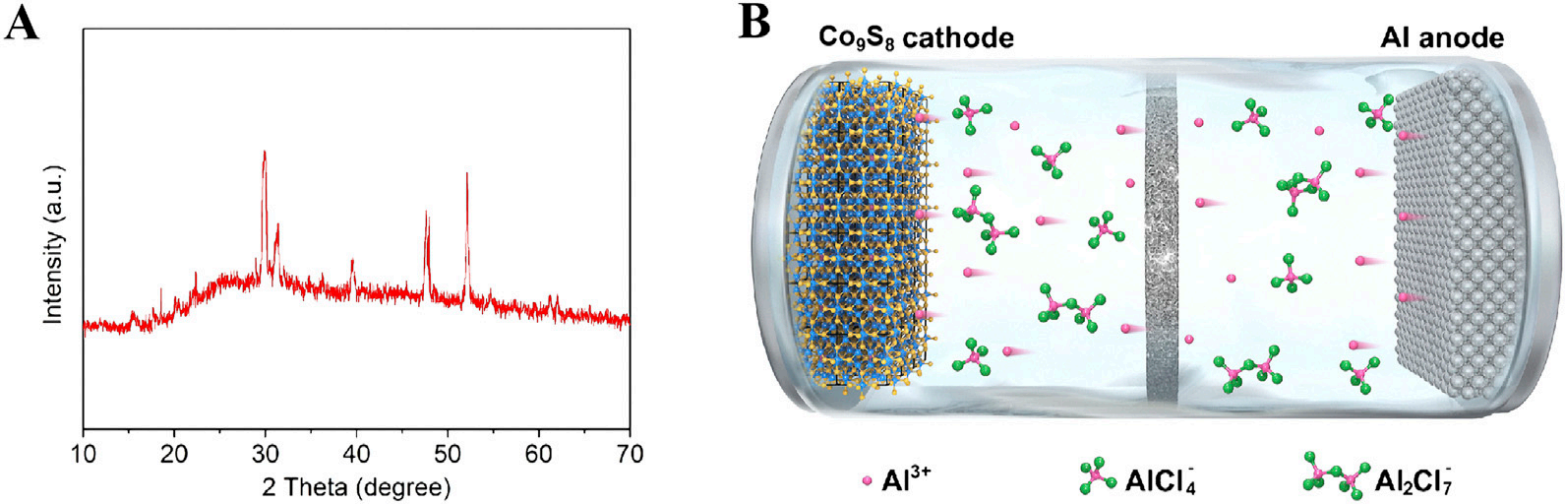
**(A)** XRD patterns of the cycled Co_9_S_8_ electrode; **(B)** Schematic diagram of charging and discharging process of Co_9_S_8_ based electrode in AIBs.

For the storage site mechanism of Al^3+^, the system considers two possible substitution mechanisms, namely, Al-Co substitution mechanism and Al-S substitution mechanism, and constructs two typical substitution models (Figures 5C,D). Al^3+^ occupies the octahedral Co. atom sites (Figure 5C), and the total energy of the crystal after substitution is −414.11 eV, with a calculated formation energy ΔEsub-Co of 0.92 eV. Figure 5D is a schematic diagram of another substitution structure of A^3+^, where Al^3+^ occupies the tetrahedral S atom sites, and the total energy of the crystal after substitution is −416.03 eV, with a formation energy ΔEsub-S of 1.98 eV. Although the formation energies obtained in both cases are within the thermodynamically acceptable range, they still belong to positive values, indicating the presence of resistance in the process. By comparing the formation energy, a lower formation energy indicates that the reaction of Al substituting for Co atoms is more thermodynamically favorable. Therefore, it can conclude that aluminum is more likely to replace Co in the Co_9_S_8_ electrode system, and the charge-discharge mechanism of the Co_9_S_8_ electrode system for AIBs is also more likely to be the mechanism of Al ion substitution reaction. To evaluate the structural stability, XRD analysis was performed on the cycled electrode (Figure 6A). Comparison reveals that the XRD pattern of the cycled electrode is largely unchanged from that of the pristine material (Figure 2A), with the exception of reduced intensity in certain diffraction peaks. This indicates excellent structural integrity of the electrode material after multiple cycles. The charge-discharge process of Co_9_S_8_ electrode system can be summarized as shown in Figure 6B.

This study reveals the capacity limiting factors and energy storage mechanism of Co_9_S_8_-based electrodes through a deep combination of experimental characterization and theoretical simulation, providing a clear direction for subsequent material design. Based on the above mechanism, in the future, it is possible to design gradient solid solutions, such as using transition metals such as Fe and Ni to partially replace Co. to form (Co.,M)9S8 solid solutions, and expand the size of ion channels through lattice regulation. This integrated experimental-theoretical approach provides critical insights into the performance limitations of Co_9_S_8_-based electrodes, guiding rational design strategies for advanced AIBs cathode materials.

## 4 Conclusion

This study successfully synthesized Co_9_S_8_ nanoparticles via a one-step hydrothermal method, and systematically investigated their energy storage mechanism and performance-limiting factors in AIBs. Structural characterization revealed that the synthesized material formed micron-sized agglomerates consisting of interconnected primary nanoparticles. XPS analysis demonstrated the coexistence of mixed Co^2+^/Co^3+^ valence states on the material surface, accompanied by significant charge delocalization in the S 2p orbital, which collectively established multiple electron transfer pathways at the electrode-electrolyte interface. Electrochemical tests showed that although the 3D ionic channels of the Co_9_S_8_ cubic phase are theoretically beneficial for the transport of Al^3+^, the material agglomeration morphology leads to significant interfacial charge transfer resistance (Rct = 128 Ω) and electrode polarization (ΔE = 0.69 V), ultimately limiting its discharge specific capacity to 48 mAh g^−1^ at 100 mA g^−1^ over 500 cycles. Density functional theory (DFT) calculations revealed a “pseudo-isomorphic substitution” mechanism, where Al^3+^ ions preferentially occupy cobalt lattice sites with a favorable substitution energy of 0.92 eV. The comprehensive investigation electrochemical behavior and pseudo-isomorphic substitution mechanism of Co_9_S_8_ based cathode establishes an atomic-scale theoretical framework for rational electrode design in AIBs. In the future, the capacity can be improved focus on synergistic engineering approaches. For example, optimizing crystallographic orientation to enhance the diffusion kinetics of Al^3+^ through low-energy migration and regulating electronic structure through anion/cation co doping to promote multi-electron redox reactions.

## Data Availability

The original contributions presented in the study are included in the article/supplementary material, further inquiries can be directed to the corresponding authors.
